# mGluR5 antagonism inhibits cocaine reinforcement and relapse by elevation of extracellular glutamate in the nucleus accumbens via a CB1 receptor mechanism

**DOI:** 10.1038/s41598-018-22087-1

**Published:** 2018-02-27

**Authors:** Xia Li, Xiao-Qing Peng, Chloe J. Jordan, Jie Li, Guo-Hua Bi, Yi He, Hong-Ju Yang, Hai-Ying Zhang, Eliot L. Gardner, Zheng-Xiong Xi

**Affiliations:** 1 Department of Psychiatry, School of Medicine, University of California San Diego, La Jolla, CA 92093 USA; 2 Molecular Targets and Medications Discovery Branch, Intramural Research Program, National Institute on Drug Abuse, Baltimore, MD 21224 USA; 3 Present Address: Psychiatry Residency Training Program, Department of Behavioral Health, Saint Elizabeths Hospital, 1100 Alabama Ave. SE, Washington, DC 20032 USA

## Abstract

Metabotropic glutamate receptor 5 (mGluR5) antagonism inhibits cocaine self-administration and reinstatement of drug-seeking behavior. However, the cellular and molecular mechanisms underlying this action are poorly understood. Here we report a presynaptic glutamate/cannabinoid mechanism that may underlie this action. Systemic or intra-nucleus accumbens (NAc) administration of the mGluR5 antagonist 2-methyl-6-(phenylethynyl)-pyridine (MPEP) dose-dependently reduced cocaine (and sucrose) self-administration and cocaine-induced reinstatement of drug-seeking behavior. The reduction in cocaine-taking and cocaine-seeking was associated with a reduction in cocaine-enhanced extracellular glutamate, but not cocaine-enhanced extracellular dopamine (DA) in the NAc. MPEP alone, when administered systemically or locally into the NAc, elevated extracellular glutamate, but not DA. Similarly, the cannabinoid CB1 receptor antagonist, rimonabant, elevated NAc glutamate, not DA. mGluR5s were found mainly in striatal medium-spiny neurons, not in astrocytes, and MPEP-enhanced extracellular glutamate was blocked by a NAc CB1 receptor antagonist or N-type Ca^++^ channel blocker, suggesting that a retrograde endocannabinoid-signaling mechanism underlies MPEP-induced glutamate release. This interpretation was further supported by our findings that genetic deletion of CB1 receptors in CB1-knockout mice blocked both MPEP-enhanced extracellular glutamate and MPEP-induced reductions in cocaine self-administration. Together, these results indicate that the therapeutic anti-cocaine effects of mGluR5 antagonists are mediated by elevation of extracellular glutamate in the NAc via an endocannabinoid-CB1 receptor disinhibition mechanism.

## Introduction

Drug addiction is a chronic brain disease characterized by drug-induced euphoria, craving and relapse to drug-seeking behavior after abstinence. There are no approved medications available for the treatment of cocaine addiction. Recent studies suggest that the metabotropic glutamate receptor 5 (mGluR5) is involved in cocaine reward and addiction^1,2^, as genetic deletion or pharmacological blockade of mGluR5 inhibits cocaine self-administration^1,3–8^ and cocaine-induced reinstatement of drug-seeking behavior^7–11^. These data suggest that mGluR5 could be an important target in medication development for the treatment of cocaine addiction. However, the cellular and molecular mechanisms through which mGluR5 modulates cocaine-taking and cocaine-seeking behaviors are poorly understood.

It is well documented that cocaine self-administration and reinstatement of drug-seeking behaviors are associated with cocaine-induced increases in extracellular dopamine (DA) and glutamate in the nucleus accumbens (NAc)^7,8,12–14^, which subsequently activates postsynaptic DA (D1-like, D2-like) and glutamate receptors^2,15–19^. Glutamate receptors include ionotropic (NMDA, AMPA, kainate) and metabotropic glutamate receptors (mGluR1-mGluR8). It is unclear how selective blockade of the mGluR5 subtype, without interruption of DA receptors and many other glutamate receptor subtypes, robustly inhibits cocaine self-administration and reinstatement of cocaine-seeking behaviors.

mGluR5s are found mainly in striatal medium-spiny neurons (MSNs)^20–23^. mGluR5 stimulation activates phospholipase C (PLC), resulting in the generation of inositol triphosphate (IP_3_) and diacylglycerol (DAG), which activates protein kinase C (PKC)^24^. Microinjections of the mGluR1/5 agonist DHPG^18^ or the selective mGluR5 agonist CHPG^10^ into the NAc reinstates cocaine-seeking behavior, while microinjections of a PLC or PKC inhibitor attenuate cocaine-induced reinstatement^18,25^, suggesting an important role of NAc mGluR5 in reinstatement of drug-seeking. However, the neural mechanisms by which mGluR5s in MSNs modulate cocaine-taking and cocaine-seeking behaviors are unknown.

*In vitro* electrophysiological evidence indicates that mGluR5 activation produces short- or long-term *depression* of glutamate release from neuronal terminals in the striatum via a retrograde endocannabinboid (eCB) mechanism. Specifically, stimulation of mGluR5 results in eCB release from postsynaptic MSN neurons, with released eCB then diffusing in retrograde fashion to activate presynaptic CB1 receptors and inhibit Ca^++^-dependent vesicular glutamate release in the NAc and the dorsal striatum^26–29^. However, little is known whether this retrograde eCB mechanism underlies mGluR5 function at behavioral level *in vivo*. Here we hypothesized that mGluR5s may modulate cocaine self-administration and reinstatement of cocaine-seeking behaviors by regulating presynaptic glutamate release via a retrograde eCB-CB1 receptor mechanism. To test this hypothesis, we used multiple animal models of drug self-administration, combined with *in vivo* brain microdialysis and transgenic CB1 receptor knockout approaches, to study the interaction of cocaine, mGluR5 and CB1 receptors on drug-taking and drug-seeking behavior and on extracellular DA and glutamate levels in the NAc.

## Results

### MPEP inhibits cocaine self-administration and cocaine-induced reinstatement of drug-seeking behavior

**Figure 1:**
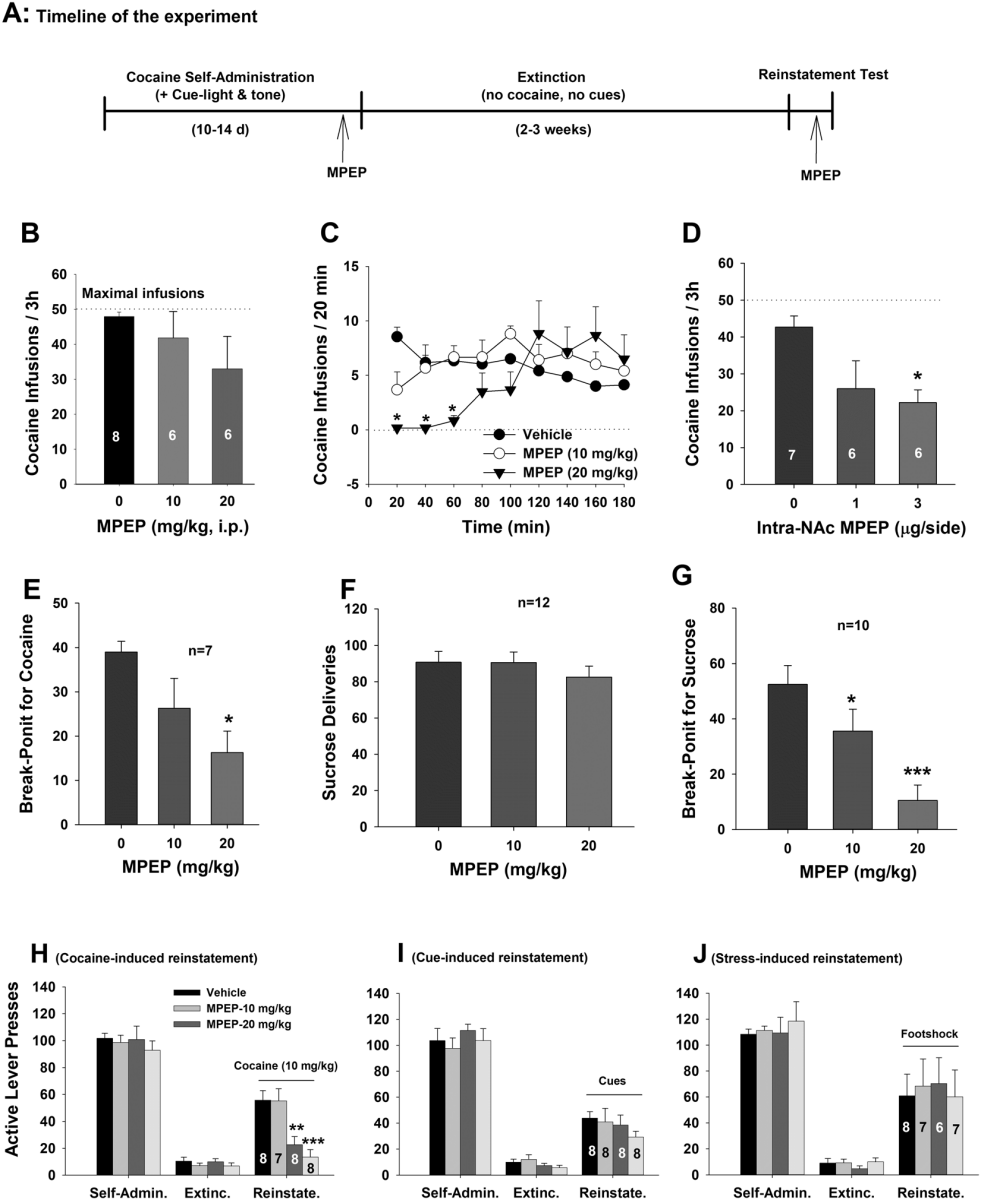
Effects of the mGluR5 antagonist MPEP on cocaine self-administration and reinstatement of drug-seeking behavior in rats. (**A**) Diagram showing the general experimental procedures. (**B**) Effects of systemic administration of MPEP (10, 20 mg/kg) on the total number of cocaine infusions during daily 3 h session; (**C**) Time course of cocaine self-administration within daily 3 h session, illustrating that MPEP significantly inhibited cocaine self-administration in the first hour of self-administration sessions. (**D**) Intra-NAc local administration of MPEP also inhibited cocaine self-administration. (**E**) Systemic administration of MPEP dose-dependently lowered the break-point for cocaine self-administration under PR reinforcement. (**F**/**G**) Systemic administration of MPEP failed to alter oral sucrose self-administration under FR2 reinforcement (**F**) but decreased the break-point for sucrose self-administration under PR reinforcement (**G**). (**H/I/J**) Systemic administration of MPEP dose-dependently inhibited reinstatement of drug-seeking behavior triggered by cocaine (**H**), but not by cues (**I**) or footshock stress (**J**). *p < 0.05, **p < 0.01, ***p < 0.001, compared to vehicle control group.

We have recently reported that the mGluR5 antagonists MTEP, fenobam and MFZ 10−7 are effective in attenuation of cocaine self-administration and relapse to drug-seeking behavior in rats^7,8^. In this study, we used MPEP, a prototypic mGluR5 antagonist^30^, to systemically evaluate its effects on cocaine self-administration and reinstatement of drug-seeking behavior. Figure 1A shows the timeline of this experiment. After cocaine self-administration was stabilized, the experimental animals were divided into three dose groups to observe the effects of MPEP on cocaine self-administration. Figure 1B shows that pretreatment with MPEP (10, 20 mg/kg, i.p., 30 min prior to self-administration) did not significantly decrease the total number of cocaine infusions within the daily 3-h session under a fixed-ratio 2 (FR2) reinforcement condition (one-way ANOVA, F_2,17_ = 1.23, *p* > 0.05). However, when we examined the time courses of cocaine self-administration during the 3-h session, we found that MPEP significantly inhibited cocaine self-administration only within the first hour of testing (Fig. 1C, two-way ANOVA with repeated measures for time: treatment, F_2,17_ = 12.72, *p* < 0.001, time, F_2,34_ = 0.61, *p* > 0.05, treatment × time interaction, F_4,34_ = 3.57, *p* < 0.05). After completing the first drug testing, the rats were continually trained for cocaine self-administration until responding was re-stabilized. Then the animals were randomly divided into three dose groups again to observe the effects of intra-NAc microinjections of MPEP on cocaine self-administration. Figure 1D shows that intra-NAc local administration of MPEP (vehicle, 1, 3 μg/side) also dose-dependently inhibited cocaine self-administration (F_2,16_ = 3.97, *p* < 0.05).

To determine whether blockade of mGluR5 alters the rewarding strength of cocaine, we used cocaine self-administration procedures under a progressive-ratio (PR) reinforcement schedule to examine the effects of MPEP on the break-point level, an index of reward strength^31^, for cocaine self-administration in an additional group of rats. Figure 1E shows that systemic administration of MPEP significantly and dose-dependently lowers the break-point for cocaine self-administration (F_1,18_ = 4.65, *p* < 0.05), suggesting reduced cocaine reward after mGluR5 blockade^31^. This reduction in cocaine self-administration is not likely due to MPEP-induced sedation or nonspecific locomotor impairment since MPEP, at the same doses as used in cocaine self-administration, failed to alter oral sucrose self-administration under FR2 reinforcement (Fig. 1F), but decreased the break-point level for sucrose self-administration under PR reinforcement (Fig. 1G).

We then examined the effects of MPEP on reinstatement of drug-seeking behavior triggered by cocaine priming, re-exposure to cocaine-associated cues, or footshock stress in additional groups of rats. The initial cocaine self-administration under FR1 and FR2 reinforcement was the same as described above. After responding was stabilized, rats underwent the extinction phase, during which lever presses did not produce any consequence, i.e., no cocaine infusions, no cocaine-associated cue lights and no tones were presented. After stable extinction criteria were met (defined as ≤10 active lever presses during three consecutive 3-h sessions), the animals were divided into three groups to observe the effects of MPEP on reinstatement responding produced by three different triggers. Strikingly, pretreatment with MPEP (vehicle, 10, 20, 30 mg/kg, 30 min prior to testing) selectively inhibited cocaine-induced reinstatement of drug-seeking behavior in a dose-dependent manner (Fig. 1H, F_3,27_ = 10.04, *p* < 0.001), but had no effect on cue-induced reinstatement (Fig. 1I, F_3,28_ = 0.28, *p* > 0.05) or footshock stress-induced reinstatement (Fig. 1J, F_3,24_ = 0.39, *p* > 0.05).

### MPEP attenuates cocaine-enhanced extracellular NAc glutamate, but not DA

**Figure 2:**
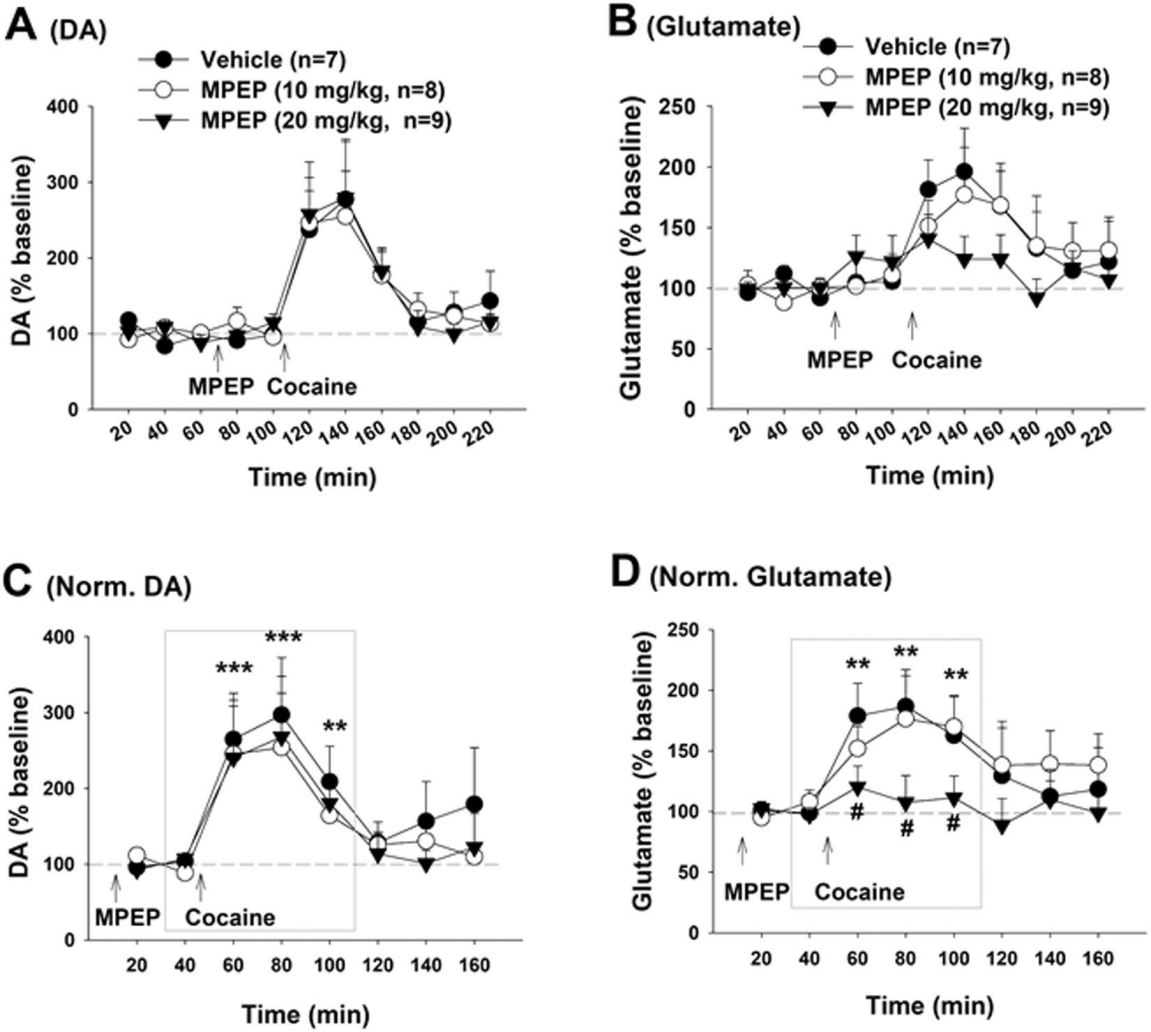
Effects of MPEP on cocaine-induced changes in extracellular DA and glutamate in the NAc. (**A**,**B**) Effects of MPEP and cocaine on extracellular DA (**A**) and glutamate (**B**) in the NAc in rats after cocaine self-administration and extinction. (**C**,**D**) Cocaine-induced % changes in extracellular DA or glutamate over new baseline (mean value of two samples before cocaine injection) in the presence or absence of MPEP, illustrating that MPEP pretreatment significantly attenuated cocaine-enhanced glutamate, but not DA, in a dose-dependent manner. **p < 0.01, ***p < 0.001, compared to pre-cocaine baseline; #*p* < 0.05, compared to vehicle control group. The inserted boxes in Panels (C,D) show the areas from which the data were subjected to statistical analysis.

We next carried out brain *in vivo* microdialysis in additional groups of rats with the same history of cocaine self-administration and extinction training, to measure cocaine-induced changes in extracellular DA and glutamate in the NAc in the absence or presence of MPEP pretreatment. Figure 2 shows that cocaine (10 mg/kg, i.p.) priming significantly increased extracellular NAc DA (Fig. 2A,C) and glutamate (Fig. 2B,D). Since MPEP pretreatment altered basal levels of extracellular glutamate before cocaine priming injections (Fig. 2B), we renormalized the data over new baselines taken immediately before cocaine injections (Fig. 2C,D). We found that MPEP pretreatment selectively attenuated cocaine-induced increases in glutamate (Fig. 2D, F_2,21_ = 4.15, *p* < 0.05), but not in DA (Fig. 2C, F_2,21_ = 0.82, *p* > 0.05), suggesting that a glutamate mechanism may underlie MPEP antagonism of cocaine-taking and cocaine-seeking behavior.

### MPEP alone increases extracellular glutamate, but not DA

**Figure 3:**
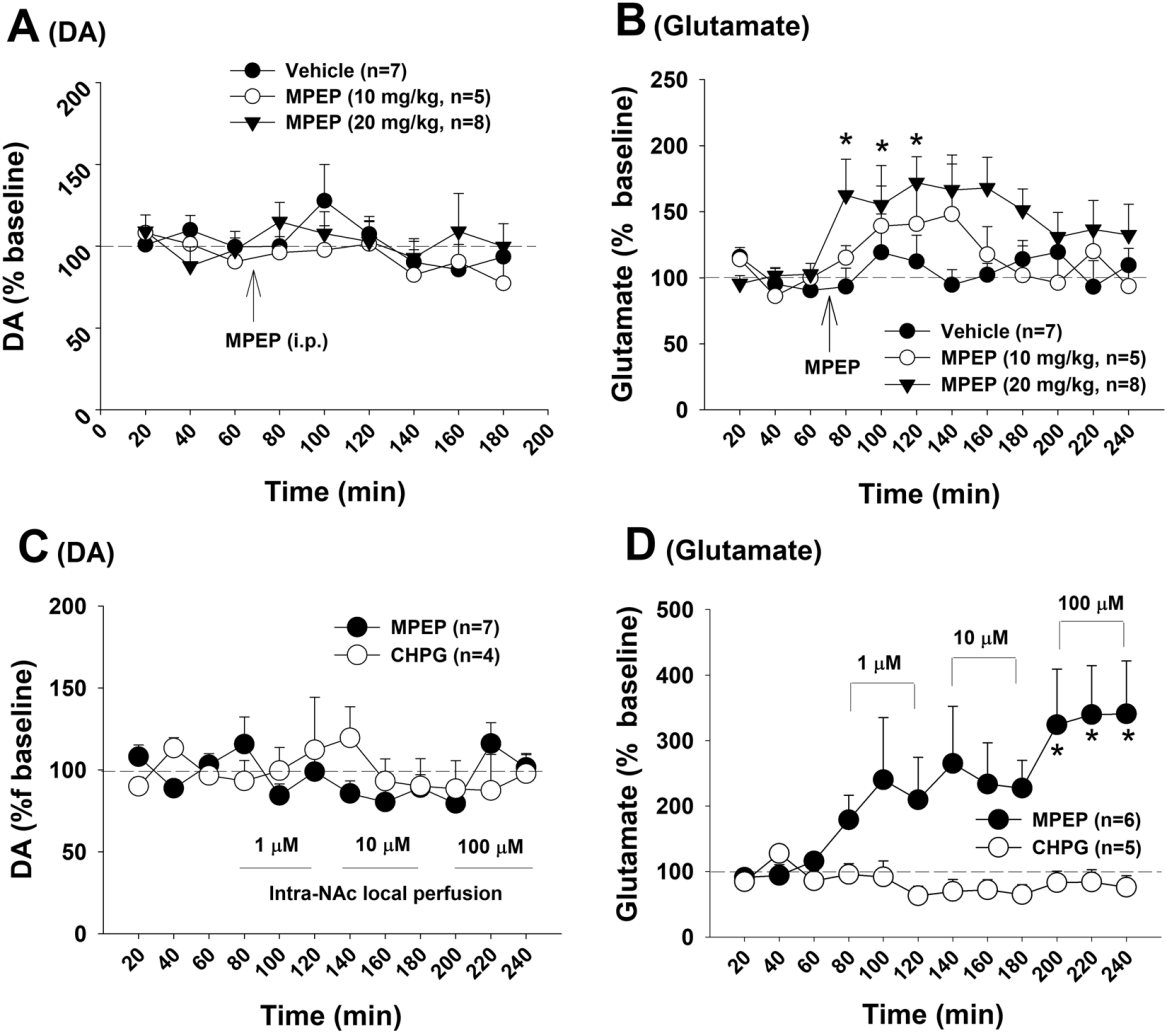
Effects of MPEP alone on basal extracellular DA and glutamate in the NAc in rats. (**A**,**B**) Systemic administration of MPEP did not alter basal extracellular DA, but significantly elevated extracellular glutamate. (**C**,**D**) Intra-NAc local perfusion of MPEP failed to alter extracellular DA, but elevated extracellular glutamate in a concentration-dependent manner. Intra-NAc local perfusion of CHPG did not produce significant changes in DA or glutamate. *p < 0.05, compared to pre-treatment baseline.

We next examined the effects of MPEP alone on extracellular DA and glutamate in the NAc. Figure 3(A,B) shows that systemic administration of MPEP failed to alter extracellular NAc DA (Fig. 3A: F_2,14_ = 1.76, *p* > 0.05), but dose-dependently elevated extracellular levels of glutamate (Fig. 3B, F_2,17_ = 3.63, *p* < 0.05). Figure 3(C,D) shows that local administration of MPEP into the NAc similarly elevated extracellular glutamate (Fig. 3D, F_11,55_ = 3.04, *p* < 0.01), but not DA (Fig. 3C: F_11,66_ = 1.72, *p* > 0.05). Surprisingly, intra-NAc local perfusion of (R,S)-2-chloro-5-hydroxyphenylglycine (CHPG), a selective mGluR5 agonist, did not significantly alter extracellular levels of DA (Fig. 3C: F_11,66_ = 1.12, *p* > 0.05) or glutamate (Fig. 3D: F_11,44_ = 0.29, *p* > 0.05).

### MPEP-enhanced extracellular glutamate is Ca^++^ channel-dependent

**Figure 4:**
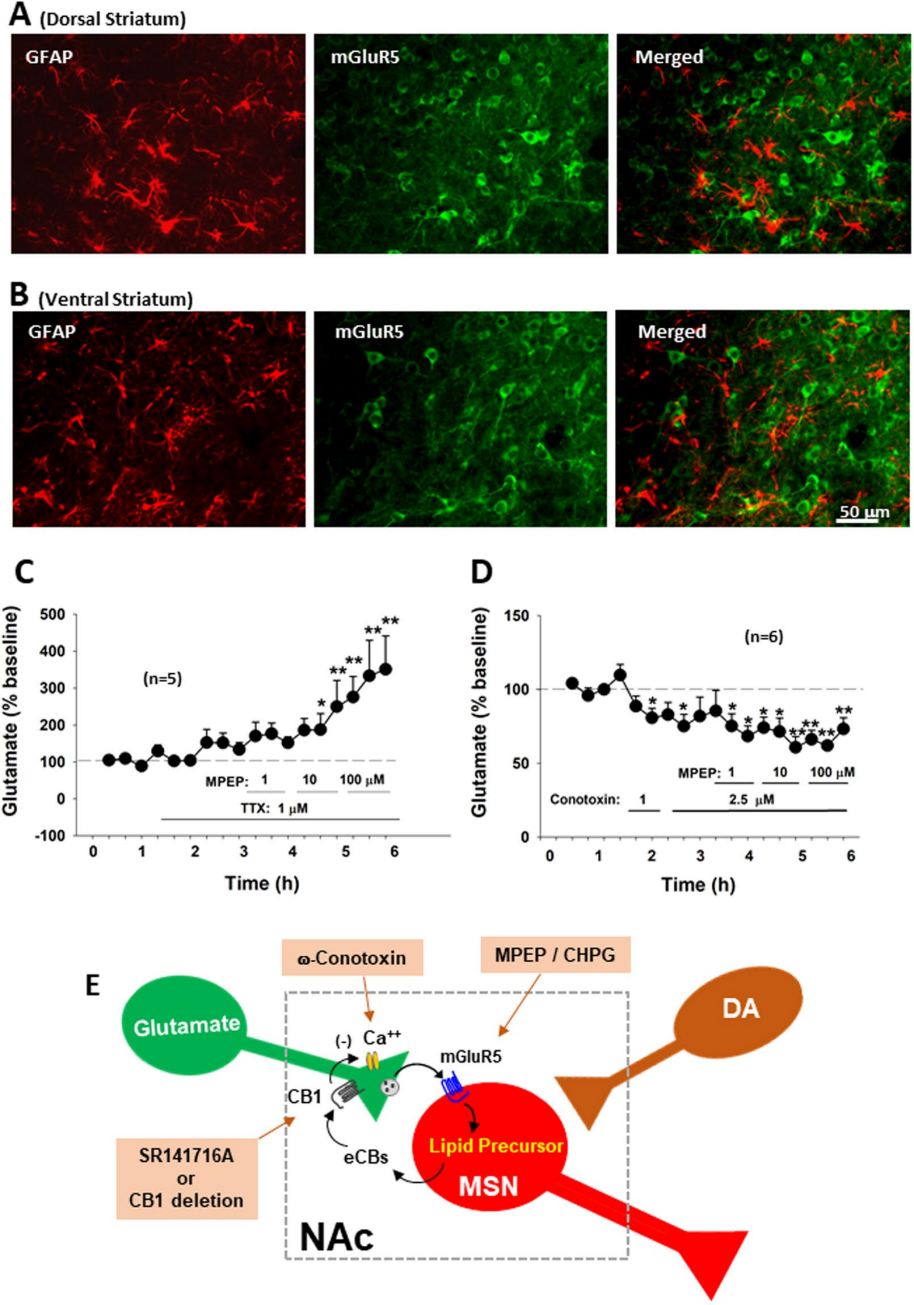
Neural mechanisms underlying MPEP-enhanced extracellular glutamate in the NAc. (**A**,**B**) The cellular distributions of mGluR5s, illustrating that mGluR5-immunostaining was detected mainly in striatal neurons, not in GFAP-labeled astrocytes. (**C**,**D**) Intra-NAc local perfusion of TTX had no effect, while ω-conotoxin GVIA blocked MPEP-enhanced extracellular glutamate. TTX alone failed to alter extracellular glutamate, while ω-conotoxin GVIA alone significantly lowered extracellular glutamate. (**E**) Diagram showing the retrograde eCB-CB1 receptor mechanism underlying MPEP-enhanced glutamate release and the targets of the pharmacological agents used in this study. *p < 0.05, **p < 0.01, compared to pre-treatment baseline.

How does mGluR5 antagonism elevate extracellular glutamate? To address this question, we first examined the cellular distributions of mGluR5 in the striatum. We found that mGluR5-immunostaining was detected mainly in cell bodies of striatal neurons, not in GFAP-labeled astrocytes (Fig. 4A,B), suggesting that mGluR5s are located mainly on postsynaptic medium-spiny neurons in the NAc and MPEP-enhanced glutamate release may be derived from neuronal, not glial, sources.

To further explore this possibility, we examined the effects of tetrodotoxin (TTX, a voltage-dependent Na^+^ channel blocker) or ω-conotoxin GVIA (a N-type Ca^++^ channel blocker) on MPEP-enhanced extracellular glutamate. Figure 4C shows that intra-NAc local perfusion of TTX (1 μM) did not alter basal or MPEP-enhanced glutamate. In contrast, intra-NAc local perfusion of ω-conotoxin GVIA (1, 2.5 μM) modestly lowered basal levels of extracellular glutamate by itself, and co-administration of ω-conotoxin GVIA blocked MPEP-induced increases in NAc glutamate (Fig. 4D). These findings suggest that MPEP-enhanced extracellular glutamate is neuronal terminal Ca^++^ channel-dependent, but not action potential-dependent.

### Presynaptic CB1 receptors modulate glutamate release

**Figure 5:**
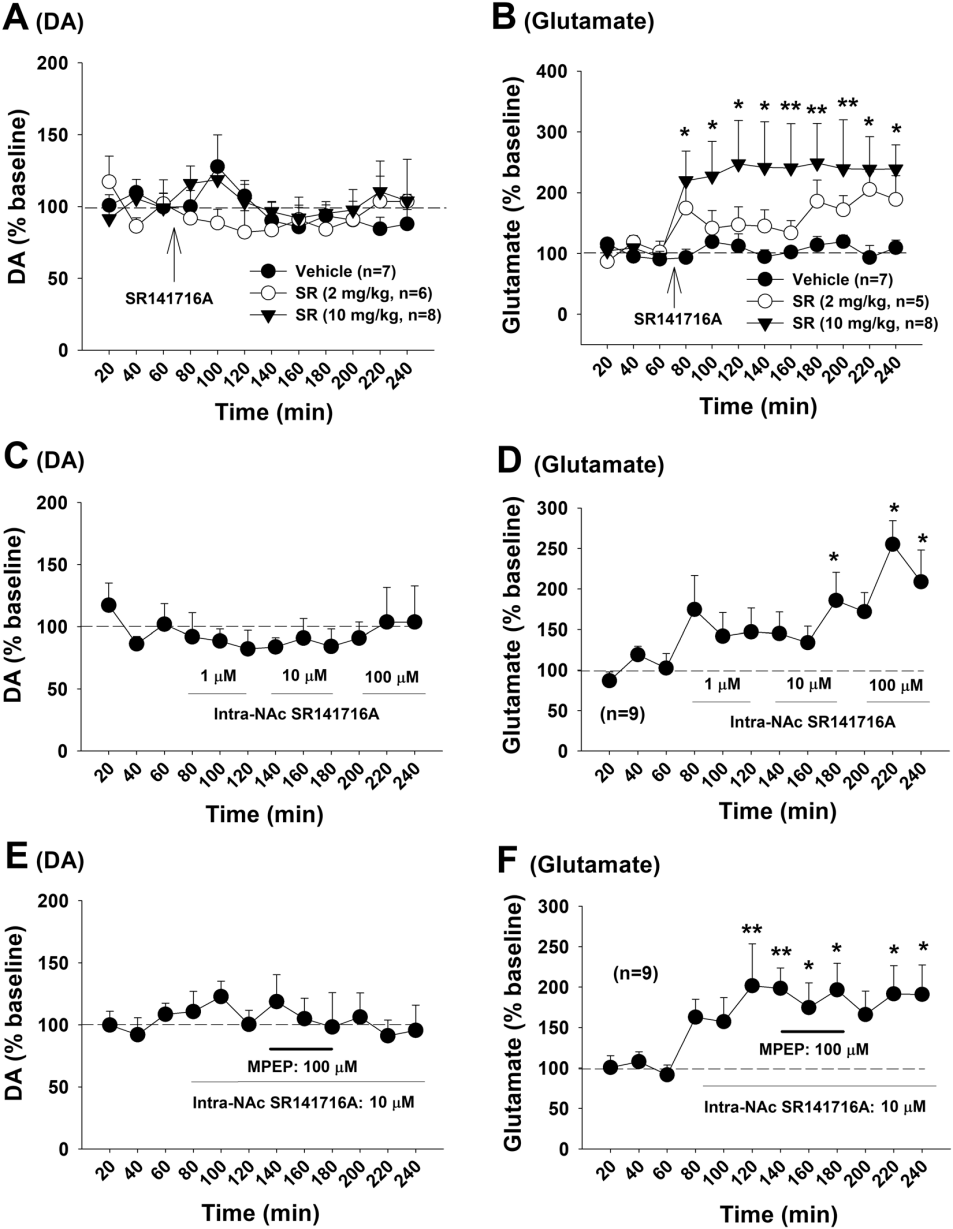
Effects of the CB1 receptor antagonist SR141716A on basal extracellular DA and glutamate in the NAc. (**A**,**B**) Systemic administration of SR141716A did not alter basal extracellular DA, but significantly elevated extracellular glutamate. (**C**,**D**) Intra-NAc local perfusion of the selective CB1 antagonist SR141716A also failed to alter extracellular DA, but significantly elevated extracellular glutamate. (**E**,**F**) Intra-NAc local perfusion of SR141716A prior to MPEP failed to alter NAc DA (**E**), but blocked MPEP-enhanced extracellular glutamate (**F**). *p < 0.05, **p < 0.01, compared to pre-SR141716A baseline.

How does postsynaptic mGluR5 antagonism facilitate presynaptic glutamate release in a Ca^++^ channel-dependent manner? As stated above, stimulation of postsynaptic mGluR5s in the striatum may inhibit presynaptic glutamate release via a retrograde endocannabinoid mechanism, which is Ca^++^ channel-dependent, but not Na^+^-channel or action potential-dependent (Fig. 4E)^26,27,29,32^. We therefore hypothesized that this retrograde eCB-CB1R mechanism may mediate MPEP-induced increases in glutamate release (Fig. 4E). To test this hypothesis, we examined whether blockade of CB1 receptors produced a similar effect as MPEP did under the same experimental conditions. Figure 5 shows that systemic administration of the CB1 receptor antagonist SR141716A (rimonabant) (2, 10 mg/kg, i.p.) also failed to alter extracellular DA (Fig. 5A, F_2,17_ = 1.02, *p* > 0.05), but significantly increased extracellular glutamate (Fig. 5B, F_2,17_ = 4.85, *p* < 0.05). Consistent with these findings, intra-NAc local perfusion of SR141716A produced the same effects as systemic administration of MPEP, that is, no effect on extracellular DA (Fig. 5C) but an increase in extracellular glutamate (Fig. 5D, F_11,88_ = 3.95, *p* < 0.001). To further determine whether MPEP-induced increases in glutamate release is mediated by inactivation of CB1 receptors, SR141716A was locally and continuously perfused into the NAc to block the NAc CB1 receptor and then co-administered with MPEP. We found that the presence of NAc SR141716A completely blocked MPEP-induced increases in glutamate (Fig. 5E,F). These data suggest that MPEP-enhanced glutamate release is CB1R-dependent, and CB1R inactivation leads to an increase in presynaptic glutamate release.

### CB1R deletion blocks MPEP’s action in glutamate release and cocaine self-administration

**Figure 6:**
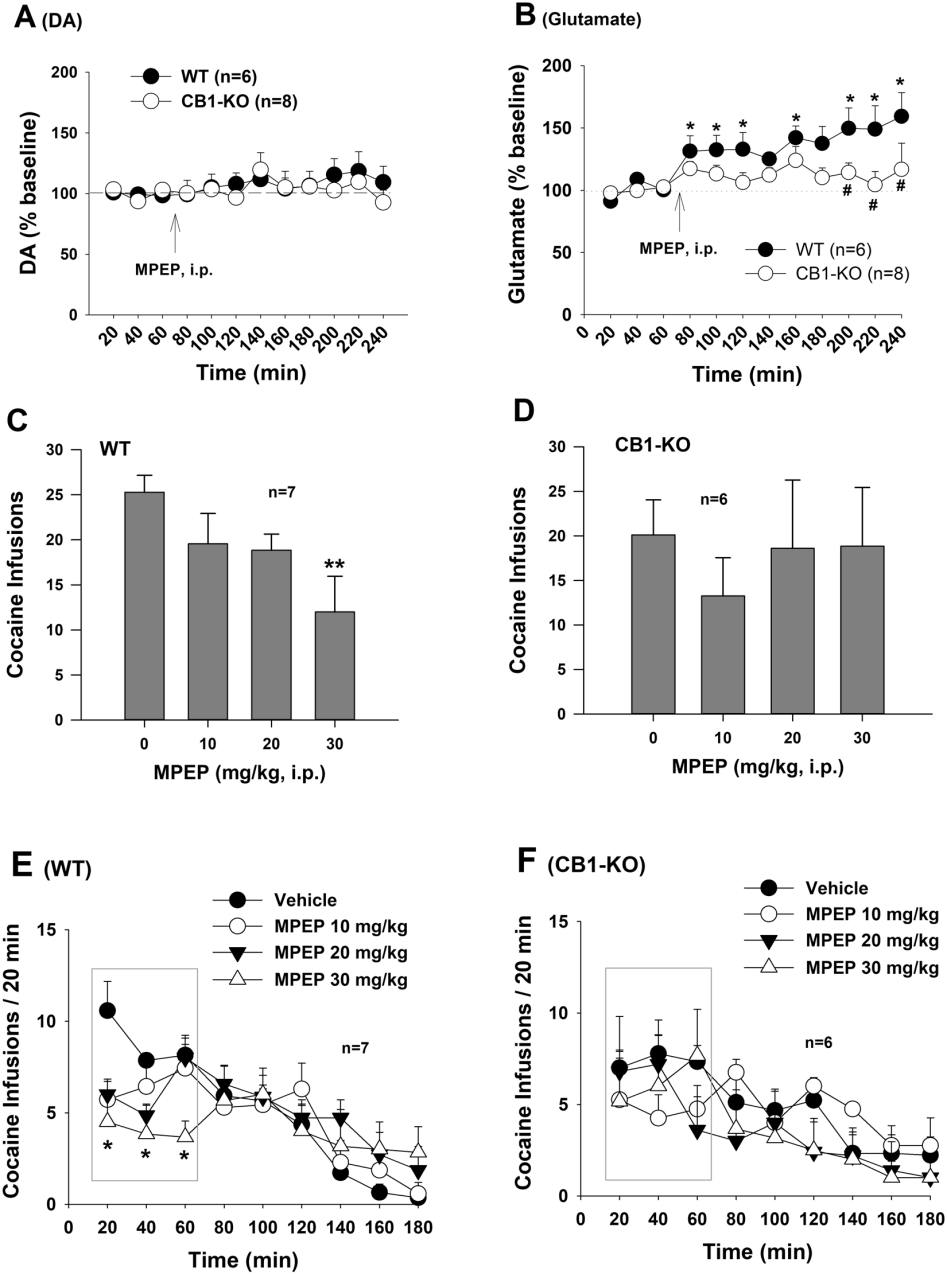
Effects of MPEP on extracellular DA and glutamate and cocaine self-administration in WT and CB1-KO mice. (**A**,**B**) Systemic administration of MPEP (10 mg/kg, i.p.) failed to alter extracellular DA in both genotypes of mice (**A**), while significantly increased extracellular glutamate in the NAc of WT mice, but not in CB1-KO mice. (**C**,**D**) Systemic administration of MPEP dose-dependently inhibited cocaine self-administration in WT mice, but not in CB1-KO mice. (**E**,**F**) Time course of mouse cocaine self-administration within daily 3 h session, illustrating that MPEP significantly inhibited cocaine self-administration in the first hour of cocaine self-administration in WT mice (**E**), but not in CB1-KO mice (**F**). *p < 0.05, **p < 0.01, compared to pre-MPEP baseline; ^#^*p* < 0.05, compared to vehicle control group (**B**).

Finally, we examined whether genetic deletion of CB1 receptors in CB1-KO mice blocks MPEP action. Figure 6 shows that systemic administration of MPEP (10 mg/kg, i.p.) did not significantly alter extracellular DA (Fig. 6A, F_1,12_ = 0.31, *p* > 0.05), but significantly elevated extracellular glutamate (Fig. 6B, F_1,12_ = 6.58, *p* < 0.05) in wild-type (WT) mice. However, in CB1-KO mice MPEP did not alter either extracellular DA or glutamate in the NAc. Similarly, systemic administration of MPEP (10, 20, 30 mg/kg) significantly and dose-dependently inhibited cocaine self-administration in WT (Fig. 6C, F_3,18_ = 4.85, *p* < 0.01) mice, but not in CB1-KO mice (Fig. 6D, F_3,15_ = 1.02, *p* > 0.05). Figure 6E shows the time courses of mouse cocaine self-administration in the absence or presence of MPEP, illustrating that the inhibitory effect of MPEP occurs mainly in the first hour of the daily 3 h session in WT mice, but not in CB1-KO mice (Fig. 6F).

## Discussion

Intravenous drug self-administration and reinstatement of drug-seeking behaviors are the most commonly used animal models for studying drug reward and relapse^33^. In the present study, we found that systemic or local administration of MPEP into the NAc significantly inhibits cocaine self-administration under FR2 and/or PR reinforcement schedules, oral sucrose self-administration under PR reinforcement, as well as cocaine-induced reinstatement of drug-seeking behaviors. This is consistent with previous reports that pretreatment with MPEP or other mGluR5 antagonists (MTEP, fenobam, MFZ 10-7) inhibit cocaine self-administration^1,3,4,6–9^ or cocaine-induced reinstatement of drug-seeking behavior^7–11,34,35^. Surprisingly, MPEP, at the same or higher doses, neither altered oral sucrose self-administration under FR2 reinforcement nor reinstatement of cocaine-seeking behavior triggered by cocaine-associated cues or footshock stress. This is different from previous reports that the mGluR5 antagonist MPEP inhibits cue-induced reinstatement of cocaine-seeking^11,34^ and that MTEP inhibits footshock-stress-induced reinstatement responding^36^. The mechanisms underlying these apparently conflicting findings are unclear. It may be related to the differences in the experimental conditions, drug doses, receptor binding affinity and other pharmacokinetic profiles of different mGluR5 ligands, or in the neural substrates underlying reinstatement of drug-seeking behaviors triggered by three distinct factors.

Whatever the reasons for the disparate findings in cue- or stress-induced reinstatement models, the focus of the present study was to understand the mechanisms by which mGluR5 antagonism inhibits cocaine self-administration or reinforcement. It is well documented that cocaine significantly elevates extracellular DA and glutamate within the NAc^13,14,37–40^, which has been thought to underlie cocaine self-administration and cocaine-induced reinstatement of drug-seeking. In the present study, we found that MPEP pretreatment failed to alter cocaine-induced increases in extracellular DA, but significantly attenuated cocaine-enhanced glutamate release, suggesting that a presynaptic glutamate mechanism may be involved. However, it is unclear how such an increase in NAc glutamate mediates cocaine reinforcement and cocaine-induced reinstatement responding.

A majority of recent studies have focused on the role of postsynaptic AMPA and NMDA receptors in relapse to drug-seeking behaviors^2,41,42^. mGluR5s are co-localized with DA D2 and NMDA receptors on striatal MSNs^43,44^ and mGluR5 activation enhances NMDA receptor-mediated responses to glutamate^24,45–47^. Therefore, one possible mechanism by which mGluR5 blockade attenuates cocaine-seeking behaviors is through reducing NMDA receptor activation by glutamate. This hypothesis was supported by one study demonstrating that mGluR5 activation produces mild depolarization and enhanced excitability of MSNs in the NAc^48^, but is not supported by another study^47^. A second possibility is that mGluR5 activation may increase AMPA receptor trafficking^42^, causing increased surface expression of GluA1-containing high-conduction AMPA receptors in the NAc^42,49,50^. In addition, mGluR5 activation increases phosphorylation of AMPA receptor GluA1 subunits^51,52^, which facilitates glutamate transmission^42^. Accordingly, mGluR5 blockade would lower AMPA receptor responses to glutamate, contributing to antagonism of cocaine-taking and cocaine-seeking behaviors. However, no direct evidence supports this conjecture.

A third possibility is the current eCB retrograde signaling hypothesis: mGluR5 activation results in eCB release, which subsequently activates presynaptic CB1 receptors and inhibits presynaptic Ca^++^-dependent vesicular glutamate release^26,29,32,53–55^. Accordingly, mGluR5 blockade should produce an increase in extracellular glutamate, which subsequently inhibits cocaine-induced glutamate release, thereby attenuating reinstatement of drug-seeking behavior. This hypothesis is supported by our findings: (1) systemic or local administration of the mGluR5 antagonist MPEP increased extracellular glutamate, not DA. (2) MPEP pretreatment dose-dependently inhibited cocaine-induced increases in extracellular glutamate and cocaine-induced reinstatement of drug-seeking behavior; (3) the CB1 receptor antagonist SR141716A alone produced an increase in glutamate similar to MPEP, and not DA. (4) Blockade of NAc CB1 receptors prevented MPEP-enhanced glutamate release (in the present study) and attenuated cocaine-enhanced glutamate release and cocaine-induced reinstatement^39^; (5) MPEP-enhanced glutamate is Ca^++^-dependent; and (6) genetic disruption of CB1 receptors blocks MPEP-enhanced extracellular glutamate and MPEP-induced inhibition of cocaine self-administration. Taken together, these findings suggest that a retrograde eCB-CB1 receptor mechanism may underlie MPEP-induced increases in extracellular glutamate and the antagonism of cocaine self-administration and cocaine-primed reinstatement.

We previously reported that blockade of CB1 receptors by AM251 or activation of mGluR7 by AMN082 elevates extracellular glutamate in the NAc, which subsequently inhibits cocaine-induced glutamate release and reinstatement of drug-seeking by activation of presynaptic mGluR2/3 autoreceptors^14,39^. Similarly, elevation of extracellular NAc glutamate by inhibition of cysteine-glutamate exchange also suppresses cocaine-induced reinstatement of drug-seeking via activation of mGluR2/3^37,41^. Therefore, we believe a fourth possibility is that this glutamate-mGluR2/3 mechanism could underlie the antagonism of MPEP on cocaine-induced reinstatement of drug-seeking behavior in the present study. Finally, rats treated chronically with cocaine display decreased basal levels of extracellular glutamate in the NAc^12,13,38,39^, which has been thought to contribute to craving and relapse to drug-seeking behavior^56^. Thus, MPEP-enhanced glutamate release may restore basal levels of extracellular glutamate in cocaine self-administering rats, and therefore, reduce drug craving and relapse to drug-seeking behavior.

We note that MPEP pretreatment selectively blocked cocaine-enhanced extracellular glutamate, but failed to alter cocaine-enhanced extracellular DA. Accordingly, MPEP pretreatment should have no significant effect on cocaine self-administration since drug self-administration is largely DA-dependent^56^. However, we have recently reported that genetic deletion of presynaptic mGluR2 autoreceptors significantly elevated extracellular glutamate, which produced a significant reduction in cocaine self-administration and cocaine reinforcement^57^. While enhanced glutamate release may increase excitability of MSNs by activation of NMDA and/or AMPA receptors^58^, enhanced DA produces an inhibitory effect on the MSNs mainly by activation of DA D2-like receptors^59^. Therefore, we propose that enhanced glutamate release may functionally antagonize DA action in postsynaptic MSNs, and subsequently attenuate cocaine self-administration under both FR2 and PR reinforcement in the present study.

Surprisingly, we found that the mGluR5 antagonist MPEP significantly increased extracellular glutamate, while the mGluR5 agonist CHPG had no significant effect, suggesting that mGluR5s are tonically activated by endogenous glutamate. This may well explain why cocaine exposure significantly blunts or eliminates mGluR5 agonist-induced long-term depression in NAc neurons^60–63^. Since cocaine exposure may increase glutamate release in the NAc, glutamate tone on mGluR5s would also be elevated and thus receptor availability to mGluR5 agonists would be reduced^64^.

In conclusion, the most important finding of the present study is that a presynaptic glutamate/CB1 mechanism may underlie the therapeutic anti-cocaine effects of mGluR5 antagonists in animal models of drug reward and relapse. Cocaine use or priming elevates extracellular NAc DA and glutamate, which subsequently activates DA (D1, D2) and glutamate (AMPA, NMDA) receptors, producing drug reward or relapse to drug-seeking behavior after cocaine abstinence. We found that mGluR5 antagonism elevates extracellular glutamate via a retrograde eCB-CB1 receptor mechanism. Enhanced glutamate, on the one hand, activates presynaptic mGluR2/3 autoreceptors and subsequently inhibits cocaine-induced glutamate release. On the other hand, enhanced glutamate may also activate postsynaptic NMDA and AMPA receptors in MSNs, which functionally antagonizes the inhibitory effect of DA on MSNs and the subsequent cocaine self-administration and relapse to drug-seeking behavior. Together, the present study provides new evidence supporting our recent finding^57^ that enhanced glutamate release in the NAc may functionally antagonizes the action produced by DA, causing a reduction in cocaine-taking and cocaine-seeking behaviors.

## Materials and Methods

### Animals

Male Long-Evans rats (Charles River, Raleigh, NC, USA) and two genotypes of mice (wild-type and CB_1_^−/−^) were used in this study. Animals were housed individually in a climate-controlled room on a reverse light–dark cycle (lights on at 1900 hours, lights off at 0700 hours) with *ad libitum* access to food and water. All experimental procedures were conducted in accordance with the *Guide for the Care and Use of Laboratory Animals* (US National Academy of Sciences), and were approved by the Animal Care and Use Committee of the National Institute on Drug Abuse of the US National Institutes of Health. The animal facility was accredited by the Association for Assessment and Accreditation of Laboratory Animal Care International.

### Experiment 1: Cocaine self-administration in rats

#### Surgery

The i.v. surgery and cocaine self-administration procedures are the same as we reported previously^14,39^. After recovery from surgery, each rat was placed into a test chamber (day time - dark phase) and allowed to lever-press for i.v. cocaine (1 mg/kg/infusion) delivered in 0.08 ml over 4.6 sec, on a fixed ratio 1 (FR1) reinforcement schedule. Each cocaine infusion was associated with presentation of a stimulus light and tone. During the 4.6 sec infusion time, additional responses on the active lever were recorded but did not lead to additional infusions. Each session lasted 3 hr. FR1 reinforcement was used for 5–7 days. Then subjects were allowed to continue cocaine (0.5 mg/kg/infusion) self-administration under FR2 reinforcement until stable cocaine self-administration was established: operationally defined as a minimum of 20 presses on the active lever per test session, less than 10% variability in inter-response interval, less than 10% variability in number of infusions taken, and less than 10% variability in number of presses on the active lever for at least 3 consecutive days. To avoid cocaine overdose, each animal was limited to a maximum of 50 cocaine injections per 3 hr session.

#### Evaluating the effects of MPEP on cocaine self-administration

We first evaluated the effects of systemic administration of MPEP (10, 20 mg/kg, i.p., 30 min prior to testing) or vehicle (saline) on FR2 cocaine self-administration in three group of rats (between-subjects design, n = 6–8/group). Effects of MPEP on cocaine self-administration were assessed by comparing mean numbers of total cocaine infusions during test sessions. Then the same groups of animals continued cocaine self-administration. After stable self-administration was achieved, the animals were divided into three dose groups again to evaluate the effects of intra-NAc microinjection of MPEP (0, 1 3 μg/side) on cocaine self-administration.

#### Evaluating the effects of MPEP on cocaine self-administration under progressive-ratio reinforcement

Initial cocaine self-administration under FR1 and FR2 reinforcement schedules was identical to that outlined above. After stable cocaine self-administration under FR2 reinforcement was established, the subjects were switched to cocaine self-administration (0.5 mg/kg/infusion) under a PR schedule, during which the work requirement of lever presses needed to receive a single i.v. cocaine infusion was progressively raised within each test session according to the following PR series: 1, 2, 4, 6, 9, 12, 15, 20, 25, 32, 40, 50, 62, 77, 95, 118, 145, 178, 219, 268, 328, 402, 492 and 603 until the break point was reached^31^. The break-point was defined as the maximal workload (i.e. number of lever presses) completed for the last cocaine infusion prior to a 1-h period during which no infusions were obtained by the animal. Animals were allowed to continue daily sessions of cocaine self-administration under PR reinforcement conditions until day-to-day variability in break point fell within 1–2 ratio increments for 3 consecutive days. Once a stable break-point was established, subjects were assigned to three subgroups to determine the effects of four different doses of MPEP (10, 20 mg/kg, i.p.) or vehicle (1 ml/kg of 25% 2-hydroxypropyl-β-cyclodextrin solution) on PR break-point for cocaine self-administration.

#### Evaluating the effects of MPEP on oral sucrose self-administration

The procedures for sucrose self-administration were identical to the procedures for cocaine self-administration except for the following: (1) no surgery was performed on the animals; (2) active lever presses led to delivery of 0.1 ml of 5% sucrose solution into a liquid food tray on the operant chamber wall along with presentation of a stimulus light and tone; and (3) each self-administration session lasted 1 hr and was capped at 100 deliveries. After stable sucrose self-administration was established, the effects of MPEP on oral sucrose self-administration under FR2 reinforcement were determined in one group of rats (*n* = 12, within-subject design). Additional group of animals (n = 10) were trained for daily sucrose self-administration first under FR2 reinforcement for 5 days and then continued for sucrose self-administration under PR reinforcement until the stable self-administration was re-established. The effects of MPEP on break-point for sucrose self-administration were assessed in this group of rats. Each animal was tested three times with different drug doses. The time intervals between testing were 2–5 days.

### Experiment 2: Reinstatement of drug-seeking behavior

After the completion of Experiment 1, animals continued cocaine self-administration until stable self-administration was reestablished. Then, the animals underwent extinction training, during which cocaine was replaced by saline and the light and sound cues that previously accompanied cocaine infusions were turned off. After drug-seeking behavior was extinguished – defined as ≤10 active lever presses during each 3 hr session for at least 3 consecutive days – the animals were divided into three groups to observe the effects of MPEP pretreatment on reinstatement of drug-seeking behavior triggered by cocaine priming, cues, or footshock stress, respectively.

#### Cocaine-induced reinstatement

On the reinstatement test day, the rats were randomly divided into three dose groups (between-subjects design, *n* = 6–8 per group). Each group of animals received vehicle, 10 mg/kg, 20 mg/kg, or 30 mg/kg MPEP (i.p.), respectively, 30 min prior to cocaine priming (10 mg/kg, i.p.). Then, the animals were placed into the operant chambers in which they previously self-administered cocaine. Reinstatement conditions were identical to those in the extinction sessions, i.e., active lever presses were recorded without drug infusions or accompanying cues for 3 hr. Effects of MPEP on cocaine-induced reinstatement were assessed by comparing mean numbers of cocaine infusions and active lever presses per test session.

#### Cue-induced reinstatement

Cue-induced reinstatement testing began 24 h after rats met the above extinction criterion^65^. During the testing, 1–2 non-contingent presentations of the cocaine-associated light and tone were given at the onset of the test session because toward the end of extinction training, most rats did not approach the lever. Subsequent lever presses then led to response-contingent deliveries of the same conditioned light-tone cues. On the test day, the animals were divided into 4 dose groups. The effects of vehicle or MPEP pretreatment (10 mg/kg, 20 mg/kg, or 30 mg/kg, i.p., 30 min prior to testing) on cue-induced reinstatement of drug-seeking behavior were evaluated.

#### Footshock stress-induced reinstatement

This experiment was conducted in additional 4 groups of rats. The initial cocaine self-administration was the same as described above. After extinction criteria were met, each group of the animals received either vehicle or one dose of MPEP (10, 20, 30 mg/kg, 30 min prior to testing). Then the rats were returned to the testing chambers and were exposed to 10 min of intermittent footshock stress (0.5 mA; 0.5 sec on; mean off period of 40 sec)^66^, delivered by a computer-controlled system. Following termination of footshock, reinstatement testing began with onset of the house light and introduction of the active and inactive levers. During each reinstatement test session, conditions were identical to those in extinction sessions. Each reinstatement test session lasted 3 hours.

### Experiment 3: ***In vivo*** microdialysis

The intracranial guide cannula implantation surgery procedures are the same as we reported previously^14,39^. The effects of MPEP and cocaine on NAc DA and glutamate were assessed in rats with the same cocaine self-administration history (1–7 days after the last cocaine self-administration session). Additional groups of rats had only intracranial guide cannulae implantation and were used to study the effects of other drugs such as MPEP, CHPG, SR141716A, TTX or ω-conotoxin GVIA alone on NAc DA and glutamate in drug naïve rats.

Additionally, to determine CB1 receptor involvement in MPEP’s action, wild-type (WT) mice and CB1-KO mice were surgically implanted with intracranial guide cannulae (MAB 4.15.IC, SciPro Inc., Sanborn, NY, USA) into the NAc. The surgery was performed under an anesthetic mixture of ketamine hydrochloride (80 mg/ml) and xylazine hydrochloride (12 mg/ml), using standard aseptic surgical and stereotaxic technique. The stereotaxic coordinates for the NAc were AP + 1.4 mm, ML ± 1.5 mm, DV −3.8 mm with an angle of 8° from vertical. The guide cannulae were fixed to the skull with dental acrylic. *In vivo* brain microdialysis was performed 5–7 days after the surgery.

#### Microdialysis procedure

The night before the experiment, concentric microdialysis probes were inserted into the NAc^14,39^. At 2 hrs after the start of perfusion, microdialysis samples were collected every 20 min into 10 µl 0.1 M perchloric acid to prevent DA degradation. Multiple doses or concentrations of MPEP or CHPG were administered alone or in combination with other drugs. Dosage ranges of the various drugs were based upon the relative EC_50_ or IC_50_ values for binding to the respective targets. MPEP, CHPG, and tetrodotoxin (TTX) were purchased from Tocris, and ω-conotoxin GVIA was obtained from Sigma-RBI. All the drugs were dissolved with filtered dialysis buffer and were freshly prepared on day of the experiment.

After collection, samples were frozen at −80 °C.

After completion of the microdialysis experiments, rats or mice were deeply anesthetized with a high dose of pentobarbital and perfused transcardially with 0.9% saline followed by 10% formalin. Brains were removed and placed in 10% formalin for histological verification of microdialysis probe locations in the brain.

#### Quantification of DA and glutamate

Dialysate DA was measured using the ESA electrochemical detection system (ESA Inc., Chelmsford, MA, USA)^14^ and dialysate glutamate was determined using HPLC with fluorometric detection^39^. The AUC of the glutamate peak was measured using an “EZChrom Elite” ESA chromatography data system. DA and glutamate values were quantified with external standard curves.

### Experiment 4: Immunohistochemistry

After the completion of reinstatement testing, a group of rats were used to examine mGluR5 expression in rats after cocaine self-administration and extinction. After brain perfusion, the brains were removed and placed in 20% sucrose phosphate buffer at 4 °C overnight. Coronal sections were cut at 10 μm on a cryostat (CM3050S, Leica Microsystems, Nussloch, Germany). Brain sections containing the striatum were blocked and floated in 5% bovine serum albumin (BSA) and 0.5% Triton X-100 phosphate buffer for 2 hr at room temperature before being incubated with primary antibodies.

IHC assays were performed using mGluR5 antibody (Millipore, Cat#: MABN 540, 1:200) and an astrocyte marker (GFAP antibody, Abcam, Cat#: ab7260, 1:500) to detect mGluR5-immunostaining in the NAc. After washes, sections were further incubated with a mixture of secondary antibodies – Alexa Fluor 488 goat anti-rabbit for mGluR5 and Alexa Fluor 568 goat anti-mouse for GFAP – in 5% BSA and 0.5% Triton X-100 phosphate buffer at room temperature for 2 hrs. Sections were then washed, mounted, and cover slipped. Fluorescent images were collected with an Olympus FV1000 Confocal System (Olympus) using manufacturer-provided software.

### Experiment 5: Cocaine self-administration in mice

Intravenous catheterization surgery and self-administration procedures were the same as described previously^67,68^. After 5–7 days of recovery from surgery, each mouse was placed into a test chamber (Med Associates, Fairfax, VT, USA) and allowed to lever-press for i.v. cocaine (0.5 mg/kg/infusion). Each lever press led to a delivery of 0.015 ml of the drug solution over 4.2 seconds under an FR1 reinforcement schedule. Each session lasted 3 hr or until the animal received the maximally allowed 50 drug infusions (to prevent drug overdose). Daily drug self-administration continued until stable day-to-day operant behavior was established with a steady behavioral response pattern for at least 3 consecutive days. The total numbers of cocaine infusions, the numbers of cocaine self-administrations during every 20-min interval, and the numbers of cocaine infusions within the first hour of the self-administration session were compared between WT and CB1-KO mice before and after MPEP administration.

### Data analysis

All data are presented as means ± SEM. One-way or two-way repeated measures ANOVA was used to determine the significance of the changes in each measure. Whenever a significant main effect was found, individual group comparisons were carried out using the Student-Newman-Keuls Method. The significance of the results was set at *p* < 0.05.
